# A network-based phenotype mapping approach to identify genes that modulate drug response phenotypes

**DOI:** 10.1038/srep37003

**Published:** 2016-11-14

**Authors:** Junmei Cairns, Choong Yong Ung, Edroaldo Lummertz da Rocha, Cheng Zhang, Cristina Correia, Richard Weinshilboum, Liewei Wang, Hu Li

**Affiliations:** 1 Department of Molecular Pharmacology and Experimental Therapeutics, Mayo Clinic College of Medicine, Rochester, MN 55905, USA

## Abstract

To better address the problem of drug resistance during cancer chemotherapy and explore the possibility of manipulating drug response phenotypes, we developed a network-based phenotype mapping approach (P-Map) to identify gene candidates that upon perturbed can alter sensitivity to drugs. We used basal transcriptomics data from a panel of human lymphoblastoid cell lines (LCL) to infer drug response networks (DRNs) that are responsible for conferring response phenotypes for anthracycline and taxane, two common anticancer agents use in clinics. We further tested selected gene candidates that interact with phenotypic differentially expressed genes (PDEGs), which are up-regulated genes in LCL for a given class of drug response phenotype in triple-negative breast cancer (TNBC) cells. Our results indicate that it is possible to manipulate a drug response phenotype, from resistant to sensitive or vice versa, by perturbing gene candidates in DRNs and suggest plausible mechanisms regulating directionality of drug response sensitivity. More important, the current work highlights a new way to formulate systems-based therapeutic design: supplementing therapeutics that aim to target disease culprits with phenotypic modulators capable of altering DRN properties with the goal to re-sensitize resistant phenotypes.

Currently, cytotoxic chemotherapeutic agents or target-specific drugs are among the major options to treat cancers in the clinic. However, existing drug-based chemotherapies fail to prevent disease relapse with stronger tumorigenicity and resistance to chemotherapeutics within one to three years. Resistance to chemotherapies is generally thought to occur as a result of *de novo* genetic variants or acquired mutations. Therefore, mutations in cancer genes have been recognized as biomarkers of drug-sensitivity. For instance, *BRAF* mutations are associated with sensitivity to BRAF, MEK1, and MEK2 inhibitors^1^, *KRAS* mutations confer resistance to anti-EGFR therapy in colorectal cancer^2^, and *BCR-ABL* rearrangement confers sensitivity to multiple ABL inhibitors^1^. Additional mutation-driven mechanisms leading to drug resistance include expression of one or more energy-dependent transporters, induction of drug metabolic enzymes, activation of DNA repair processes, suppression of apoptosis^3^ and acquisition of bypass routes in pathway activities^4^.

The identification of mutated genes, a hallmark of cancer^5^, has led to the development of a number of anticancer drugs such as imatinib and erlotinib with proven clinical success in eradicating cancer cells that harbor a specific gene mutation. Nonetheless, due to the rapidly evolving nature of cancer cells^6^, it is simply not practical to overcome drug resistance with drugs designed to alter one particular target. Cancer cells inevitably adapt to counteract the pharmacological actions of such chemotherapeutics, resulting in drug resistance. In addition, increasing clinical evidence points to the role of unstable, non-genetic mechanisms of drug resistance^7^, indicating both genetic and non-genetic factors are involved in regulating drug response phenotype and biological pathway crosstalk at the systems level. The mechanisms causing drug resistance are far more diverse than mere genetic mutations. The interplay of biological processes governing genome stability, cell proliferation, cell survival, protein synthesis, transport, and degradation can collectively determine how a cancer cell responds to a drug.

A system biology approach provides additional insights, beyond those attributable to conventional genetic and molecular biology approaches, to decipher the complex mechanisms driving a phenotype. The burgeoning field of network and systems biology offers the unprecedented opportunity for researchers to capitalize on large-scale omics data. Since 2000s, a number of network biology tools have been developed to infer regulatory and signaling circuits^8^, and disease gene candidates^9^,^10^,^11^ as well as predict drug response^12^,^13^,^14^ using protein-protein interaction (PPI) networks as a discovery platform. In addition, the International Cancer Genome Consortium (ICGC) recently provided a comprehensive overview of methodologies used to characterize pathways and networks from cancer genomes^15^.

Despite advancements in developing network-based approaches, applying systems pharmacology to clinical practice is still in its infancy. Although the use of drug combinations has been found to dramatically increase treatment efficacy and reduce incidence of drug resistance, the reduction in drug resistance is far from elimination. “Simple” drug treatments that use one or two drugs at a time for a treatment regimen are well defined in terms of pharmacological modes of action but pose higher risks of drug resistance. While current systems-based pharmacology and therapeutic designs mainly focus on finding new disease targets and novel drugs targeting them^16^,^17^, complications in developing effective therapeutics that exhibit high specificity to disease targets have limited clinical usage.

A more sustainable strategy may include sensitizing drug resistant cells to the original chemotherapeutics using systems-guided combinatorial drug treatments. This approach involves administering the original chemotherapeutics with drugs designed to alter the resistance driven by the underlying activities of drug response networks (DRNs). However, this requires knowledge of genes that constitute a DRN and which gene candidates within a DRN should be targeted to restore drug sensitivity in resistant cells. Toward this end, we developed a network-based phenotype mapping (P-Map) approach to identify gene components that constitute a DRN.

Based on the “common network-common phenotype” hypothesis^18^, drug response network inferred from one cell type can be applicable to another cell type if they share certain aspects of response network properties such as common core network motifs. Here, we used Epstein-Barr Virus (EBV)-transformed lymphoblastoid cell lines (LCLs) derived from human B-lymphocytes^19^,^20^ as a common cell-based discovery platform for the discovery of drug response networks. As a proof-of-principle study, we chose anthracyclines (doxorubicin and epirubicin) and taxanes (docetaxel and paclitaxel), since they are anticancer agents that are commonly used to treat diverse types of cancers^21^,^22^,^23^,^24^,^25^,^26^.

Using P-Map, we were able to infer response networks with respect to anthracyclines and taxanes corresponding to sensitive and resistant phenotypes. We then used triple-negative breast cancer (TNBC) cell lines to study the effects on drug responses when the expressions of selected gene candidates in DRNs are perturbed. Our results indicate that it is possible to manipulate drug response phenotypes by perturbing the activities of DRNs. The current study therefore provides a new avenue for researchers to chart drug response networks and prioritize interacting gene candidates to facilitate the development of systems-guided combinatorial drug treatments that re-sensitize resistant cancer cells.

## Results

### The design of P-Map methodology

**Figure 1:**
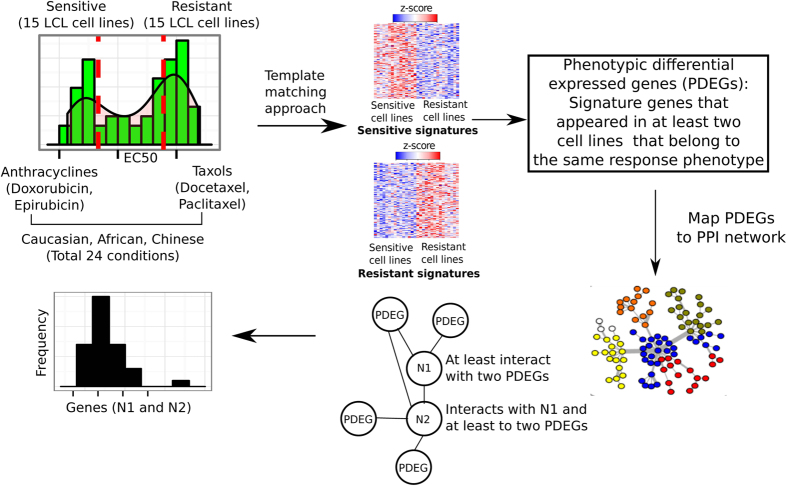
Phenotype mapping (P-Map) approach to dissect drug response networks. The concentration of anthracyclines (doxorubicin and epirubucin) and taxanes (docetaxel and paclitaxel) producing a half-maximal response (EC50), in LCL cells derived from African, Caucasian, and Chinese subjects was used. Cell lines exhibiting the most extreme responses to these drugs were defined as either sensitive or resistant. Basal transcriptomic profiles (without drug treatment) for these sensitive and resistant cell lines were retrieved using a template matching approach to identify genes that consistently show differential expression across all 15 cell lines within the same drug response condition (e.g., paclitaxel sensitive among Caucasians). Genes that show higher differential expression (i.e. up-regulated) under at least two cell lines grouped under same class of drug response phenotype (e.g., paclitaxel sensitive among Africans and docetaxel sensitive among Chinese) that belong to the same drug-response phenotype (e.g., taxane sensitive) are considered as phenotypic differentially expressed genes (PDEGs). PDEGs are then mapped onto a protein-protein interaction (PPI) network. Genes that directly interact with at least two PDEGs are termed PDEG-interacting genes. The first interacting neighbors for PDEGs do not necessarily interact with N2 PDEG-interacting genes. N2 PDEG-interacting genes have direct interaction with N1 PDEG-interacting genes and they also interact with at least two other PDEGs. These PDEGs and PDEG-interacting genes collectively constitute a drug response network (DRN). We obtained a total of 4 DRNs in this work: anthracycline-sensitive, anthracycline-resistant, taxane-sensitive, and taxane-resistant. The importance of a PDEG-interacting gene is estimated by computing the relative frequency with which they interact with PDEGs within the network.

We designed a network-based, phenotype mapping approach, termed P-Map (Fig. 1) to address the lack of tools available to dissect a biological network that is responsible for modulating cellular response to a given drug using basal transcriptomic data (i.e. prior to drug treatment). We chose anthracyclines (doxorubicin and epirubicin) and taxanes (docetaxel and paclitaxel) for our case study. We used basal transcriptomics profiles (without drug treatment) for EBV-transformed lymphoblastoid cell lines (LCLs) (GEO accession number GSE23120) obtained from African, Caucasian, and Chinese subjects as a common discovery platform. Measured sensitivity to anthracyclines and taxanes (Supplementary Data 1) was used to define drug response phenotypes (i.e., sensitive or resistant to anthracyclines or taxanes).

We considered four major drug response phenotypes in this study: sensitive and resistant based on drug class (anthracyclines or taxanes). Response phenotypes were first defined at the level of individual drugs and ethnicity. For instance, LCL cell lines derived from African origin with measured EC50 values for doxorubicin, an anthracycline, was used to identify cells that show “extreme” responses (i.e. cell lines that fall on both ends of EC50 distribution) for sensitive and resistant phenotypes for doxorubicin. Next, we measured the response of each LCL cell line derived from Caucasian and Chinese origin in respect to doxorubicin to define corresponding response phenotype. The same approach was applied to epirubicin, docetaxel, and paclitaxel in respect to each ethnic group and resulted in a total of 24 response phenotypes corresponding to each drug and ethnic group (Fig. 1).

To determine the number of cell lines to be used for the respective sensitive and resistant phenotypes, we surveyed the distribution of EC50 values for LCL cell lines of African, Caucasian, and Chinese origin in response to anthracyclines (doxorubicin and epirubicin) and taxanes (docetaxel and paclitaxel) (Supplementary Figure 1). Cells at the lower end of the EC50 distribution indicate a sensitive phenotype, whereas those at the upper end indicate a resistant phenotype. We found 15 cell lines (or samples) covered those extreme phenotypes (sensitive and resistant), while preserving a sufficient number of samples to extract meaningful information. Our subsequent analyses therefore included 15 cell lines that covered each extreme phenotype for all drugs and ethnic groups in this study.

To identify genes exhibiting differential gene expression patterns in sensitive versus resistant drug response phenotypes, which we have termed phenotypic differentially expressed genes (PDEGs), we employed a template matching method^27^. Unlike fold change methods, which are affected by the presence of outlier samples showing unusually high or low expression values, the template matching method utilizes analysis of variance to select genes showing state- or region-dependent expression patterns across samples annotated with the same biological trait. We sought to identify genes consistently exhibiting two-state expression profiles, or those genes exhibiting increased expression in a sensitive drug response phenotype but decreased expression in a resistant drug response phenotype or vice versa across cell lines using a template matching method as indicated by heatmaps show in Supplementary Data 2. The minimal criterion for a gene to be defined as phenotypically differentially expressed is that it should show upregulation in at least two cell lines in respect to any ethnic group representing the same drug response phenotypes. For instance, a PDEG in an anthracycline resistant network must exhibit higher gene expression (or up-regulation) in any two of the resistant cell lines corresponding to doxorubicin or epirubicin response in African, Caucasian, or Chinese. Such PDEGs (Supplementary Data 3) are most likely involved in biological pathways where their collective activities give rise to drug response phenotypes. We also examined to what extent inferred PDEGs from LCL-derived sensitive and resistant phenotype for taxanes with breast cancer cell lines derived from Cancer Cell Line Encyclopedia (CCLE)^28^ and the respective number of overlapped PDEGs are given in Supplementary Figure 2.

In addition, although not necessarily differentially expressed, genes that interact with multiple PDEGs can be potential modulators of drug response and should also be considered as part of the drug response network (DRN). Human protein-protein interaction (PPI) network was used as a generic network platform for the identification of DRNs. To dissect DRN corresponding to a drug response phenotype that captures global biological properties covered in a PPI network, PDEGs were used as “seeds” with each shared “PDEG-interacting gene” connecting at least two PDEGs. We therefore mapped PDEGs to PPI networks to identify genes that interacted with at least two PDEGs (Supplementary Data 4). The importance of these PDEG-interacting genes in conferring drug response was estimated by computing the frequency (called phenotypic frequency) of their interactions with PDEGs in the network (see Methods for detailed description).

### Drug response phenotype is an emergent property resulting from the interplay of biological pathways

**Figure 2:**
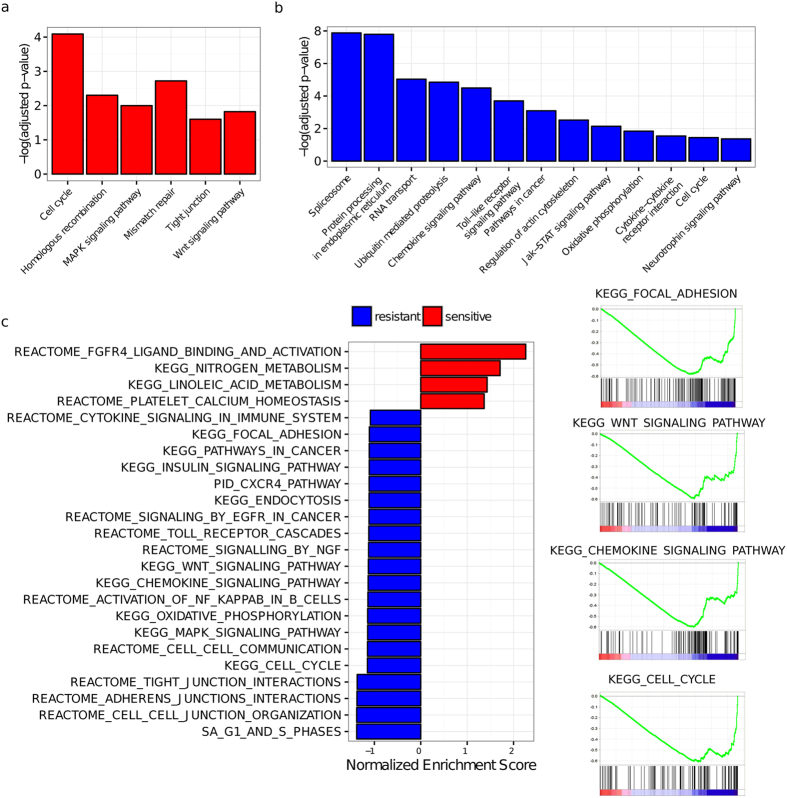
PDEG- and PDEG-interacting gene-enriched biological pathways in DRNs that confer anthracycline sensitivity and resistance. (**a**) Enriched pathways for PDEGs from anthracycline sensitive response network; (**b**) Enriched pathways for PDEGs from anthracycline resistant response network; (**c**) Enriched pathways from Gene Set Enrichment Analysis (GSEA) using pre-ranked option for PDEG-interacting genes from anthracycline sensitive and resistant response networks, ranked with phenotypic frequency (PF) scores. Red: enriched pathways from anthracycline sensitive network (positive PF scores); blue: enriched pathways from anthracycline resistant network (negative PF score). Four selected GSEA-enriched pathways from PDEG-interacting genes in anthracycline resistant network are indicated at right panel.

We performed pathway enrichment analyses to investigate which key canonical pathways were enriched for PDEGs and their interacting genes. We reasoned that these enriched pathways play important, if not direct roles, in determining drug response phenotypes. We found that enriched pathways modulating anthracycline responses included those governing the cell cycle (Cell Cycle, MAPK Signaling Pathway, Wnt Signaling Pathway, Pathways in Cancer), DNA repair (Mismatch Repair, Homologous Recombination), cell-cell interactions (Tight Junction, Regulation of Actin Cytoskeleton), biomolecular processing and transport (Spliceosome, Protein Processing in Endoplasmic Reticulum, RNA Transport, Ubiquitin-Mediated Proteolysis), cellular metabolism and regulation of cellular energetic (Oxidative Phosphorylation or OXPHOS), and immune-related responses (Chemokine Signaling Pathway, Toll-like Receptor Signaling Pathway, Jak-Stat Signaling Pathway) (Fig. 2a,b). Similar pathway categories were also found enriched for PDEGs corresponding to taxane responses (Supplementary Figure 3a,b). We next performed pathway enrichment analyses for PDEG-interacting genes using computed phenotypic frequency (PF) score, which estimates how often a given gene acts as a PDEG-interacting gene in both sensitive and resistant networks (see Methods for a detailed description). Pathways of functional categories as described for PDEGs were also found enriched for PDEG-interacting genes (Supplementary Data 5) in respect to anthracyclines (Fig. 2c) and taxanes (Supplementary Figure 3c).

Given that changes in glycosylation in tumor cells facilitate metastasis^29^, and that alterations in N-linked glycosylation in human hepatocellular carcinoma cells are associated with resistance to common anticancer agents such as epirubicin and mitoxantrone^30^, we found relevant glycosylation pathways: Protein Processing in Endoplasmic Reticulum (anthracycline-resistant PDEG enriched, Fig. 2b), Protein Processing in Endoplasmic Reticulum, N-Glycan Biosynthesis, (taxane-sensitive PDEG enriched, Supplementary Figure 3a) supporting the roles of N-glycan alterations and endoplasmic reticulum-mediated signaling in regulating drug response phenotypes.

Most importantly, we found functional coordination between PDEG- and PDEG-interacting gene-enriched pathways. For instance, the activities of oxidative phosphorylation (OXPHOS), a PDEG-enriched pathway in resistant phenotypes for both anthracyclines and taxanes (Fig. 2b, Supplementary Figure 3b), are functionally linked to energy-dependent processes such as ubiquitin-mediated proteolysis and transporter activities such as drug efflux pumps and ATP-binding cassette (ABC) transporters. Furthermore, it has been shown that cellular energetic propensity is a useful predictor for drug sensitivity and that activation of OXPHOS can promote sensitivity to anticancer agents in cancer cells^31^. In addition, glycosylating cell adhesion molecules affect cell structure and cell-cell interactions^32^ that can in turn affect cell adhesion-mediated drug response processes.

### Knocking down the expression of PDEG-interacting gene candidates altered drug responses

To determine whether perturbing the expression of PDEG-interacting genes inferred from LCLs can affect drug response in cancer cells, and to examine the effect to change of drug response. To investigate to what extent the signs (positive and negative) of computed phenotypic frequency (PF) scores predict the directionality of drug response phenotype change (i.e. enhanced or reduced drug sensitivity), we focused on anthracycline-resistant (Fig. 2c) and taxane-sensitive (Supplementary Figure 3c) phenotypes, since they exhibit more PDEG-interacting-gene-enriched biological pathways. We selected 19 genes from the anthracycline resistant DRN (Table 1) and 13 PDEG-interacting genes from the taxane sensitive DRN (Table 2) that show high, moderate, and low overall PF scores. We knocked down selected PDEG-interacting genes in triple-negative breast cancer (TNBC) cell lines (BT549 and MDA-MB-231). TNBC cells lack estrogen receptors (ER), progesterone receptors (PR), and amplification of ERBB2 (HER2)^33^, which renders TNBC patients, who typically have lower overall survival rates than patients with other types of breast cancers^34^, ineligible for hormonal therapies or HER2-targeted agents. TNBC patients are usually subjected to standard chemotherapeutic regimens using anthracyclines or taxanes^35^, which are ideal cancer models to test our hypothesis.

**Figure 3:**
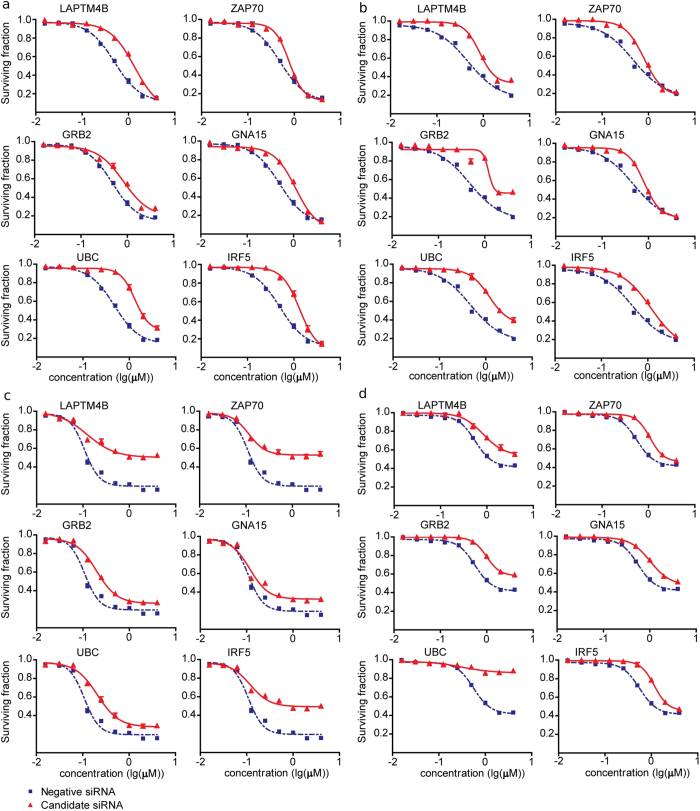
Response curves of MTS assays before and after knockdown of selected PDEG-interacting genes in BT549 and MDA-MB-231 triple-negative breast cancer (TNBC) cell lines treated with anthracyclines (doxorubicin and epirubicin). (**a**) siRNA knockdown followed by doxorubicin treatment in BT549 cells; (**b**) siRNA knockdown followed by epirubicin treatment in BT549 cells; (**c**) siRNA knockdown followed by doxorubicin treatment in MDA-MB-231 cells; (**d**) siRNA knockdown followed by epirubicin treatment in MDA-MB-231 cells. Red: candidate siRNA; blue: negative control siRNA. X-axis: drug concentrations in logarithm scale of μM; Y-axis: fraction of surviving cells. The significance of the surviving fraction between negative control siRNA and gene-specific siRNA was determined by two-tailed unpaired t-test with p-value < 0.05 is considered statistically significant.

Our results show that the majority of PDEG-interacting genes indeed altered drug response sensitivity upon RNAi knockdown (Table 1 and Table 2). However, only approximately 50% of tested PDEG-interacting genes show predicted change of drug response directionality (e.g. become more sensitive or resistant) by phenotypic frequency (PF) score. We found that knockdown of 10 of 19 PDEG-interacting genes selected from the anthracycline-resistant response network followed by MTS assay promoted resistance to doxorubicin and epirubicin in both BT549 and MDA-MB-231 TNBC cell lines (Fig. 3). In contrast, knocking down the expression of 6 of 13 PDEG-interacting genes selected from the taxane-sensitive response network enhanced sensitivity to docetaxel and paclitaxel in both BT549 and MDA-MB-231 TNBC cell lines (Supplementary Figure 4). Our results indicate it is indeed possible to manipulate drug response phenotypes by perturbing PDEG-interacting genes. It remains to be elucidated whether additional factors and plausible molecular mechanisms at the network level regulate directionality of drug responses.

## Discussion

The “war on cancer”^36^, specifically regarding resistance to cytotoxic chemotherapeutics, has mainly centered on combating genetic mutations or polymorphisms that render binding of drugs to their target ineffective; mutations that activate drug metabolizing enzymes and transporters; or mutations that bypass drug targets via alternative pathway routes. We designed P-Map to expand the battle beyond targeting mutations to explore the possibility of altering the properties of a drug response network by perturbing activities of modulatory genes in the network. As such, the P-Map algorithm differs from other network-based approaches to study drug responses. First, P-Map is designed to uncover biological networks that modulate drug response phenotype, in particular networks that sustain sensitive and resistant phenotypes. Second, unlike methods that aim to identify therapeutic targets via mutational profiles mapped onto a PPI network^13^, P-Map uses basal transcriptomics data to discover gene candidates (e.g. PDEG-interacting genes) whose expression can be perturbed to alter drug response phenotypes (e.g. re-sensitize a resistant phenotype). Compounds that interfere with protein interactions and expression of PDEG-interacting genes are potential drug response modulators that may help to maintain sensitivity of a network or destabilize a resistant network in order to enhance treatment efficacy of therapeutics. Finally, P-Map utilizes both phenotypic information (e.g. sensitivity to a drug) and basal transcriptome data (before cells are treated with a given drug). P-Map can therefore infer an inherent biological network that confers a drug response phenotype rather than the response networks generated after cells are treated with drugs^37^. The advantage of this approach is that it is possible to computationally predict a drug response phenotype before a patient receives treatment. However, P-Map is similar to some existing state-of-the-art network-based methods in its ability to identify global disease and drug response networks. In general, these methods begin with “seeds”, such as known disease or mutated genes, and use algorithms, such as network propagation^10^, flow^13^, and shortest paths^9^,^11^ to connect these seeds to generate disease and drug response networks where key gene candidates can be prioritized. For instance, the method developed by Qin *et al*., uses a flow-based algorithm to identify gene components that connect gene mutations (mutated genes as seeds) to predict network modules that modulate anticancer drug sensitivity^13^. In comparison, P-Map first infers phenotypic differentially expressed genes (PDEGs) using phenotypic and basal transcriptomics data to identify PDEG-interacting genes (PDEGs as seeds) connecting multiple PDEGs in a PPI network, thereby inferring a global drug response network.

Using a network-based approach to dissect the components of drug response networks (DRNs), the current study reveals that drug response phenotypes are shaped by the collective actions of biological pathways that support cell survival and homeostasis. In addition, it highlights the versatility of cells to acquire resistance to any kind of anticancer drug via a myriad of pathways. Our pathway enrichment results suggest that pathways participating in regulating cell survival and cellular homeostasis are key ingredients in modulating drug responses; the specific pathways falling under the cell survival and cellular homeostasis umbrellas vary depending on which drug is used. These enriched pathway categories, which represent “cancer hallmarks”^38^, suggest that the same pathway categories are also applicable in modulating drug response in cancer cells. For instance, cell cycle-mediated drug resistance was first reported at least two decades ago^39^, and processes linked to cell survival, such as DNA repair and apoptosis, are also known to play a critical role. Adhesion of small cell lung cancer cells to extracellular matrix (ECM) also has been shown to enhance tumorigenicity and resistance to chemotherapeutic agents^40^. Furthermore, cell-cell adhesion has been found to regulate resistance to the apoptotic effects induced by doxorubicin and melphalan^41^. This cell adhesion mediated drug resistance (CAM-DR) was also observed in tumor-derived endothelial cells exposed to DNA damaging agents^42^ and survival of breast cancer cells to chemotherapy such as paclitaxel^43^. The current study thus highlights the importance of considering drug response as a consequence of global, incorporated pathway activities where their relative contributions give rise to the emergent drug response phenotypes.

**Figure 4:**
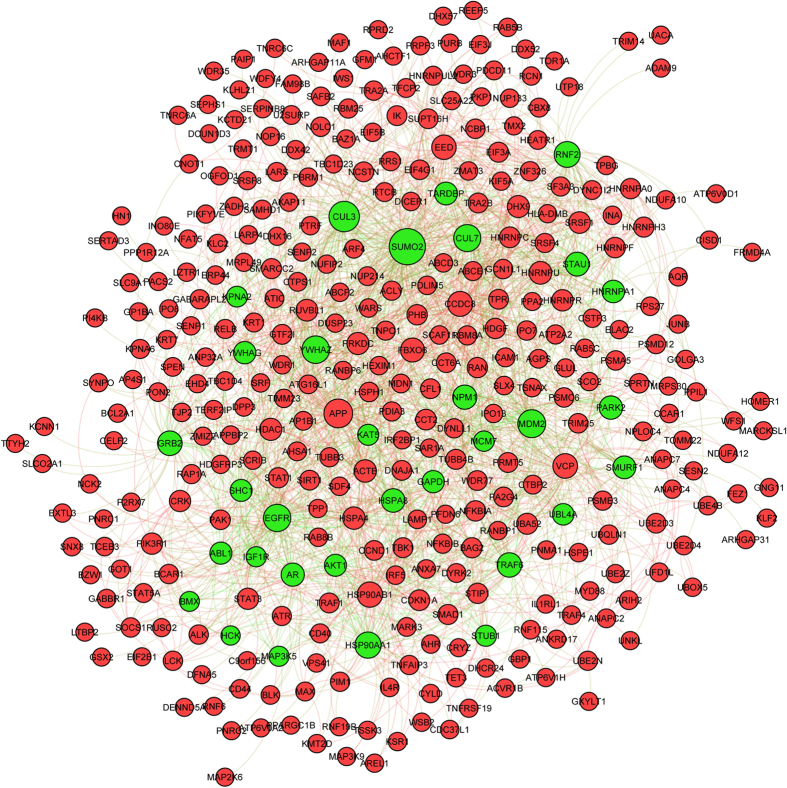
Network for anthracycline-resistant N1 PDEG-interacting genes. A portion of the anthracycline-resistant drug response network showing N1 PDEG-interacting genes (green nodes) exhibiting high PF scores and their corresponding interacting PDEGs (red nodes).

We found PDEG-interacting genes with high PF (phenotypic frequency) scores and their interacting PDEGs for anthracycline-resistant (Fig. 4) and taxane-sensitive (Supplementary Figure 5) phenotypes are densely connected (computed distributions for the degree of connectedness for these networks are given in Supplementary Figure 6). For instance, *GRB2, EGFR, MDM2, HSP90AA1*, key mediators of general signaling processes, apoptosis, and protein folding, are PDEG-interacting genes in the anthracycline-resistant response network; they interact with PDEGs such as *BCL2A1, PIK3R1, STAT1, SIRT1, CCND1*, which regulate the cell cycle, histone acetylation, and metabolism (Fig. 4). In taxane-sensitive response networks, *SIRT1, MYC, MAPK1, CDK2*, and *MDM2*, key genes in regulating the cell cycle, transcription, general signaling processes and apoptosis, interact with PDEGs such as *TAF2, SHC1, HSP90AA1, CASP8*, which regulate transcription, general signaling processes, protein folding, and apoptosis (Supplementary Figure 5). Such interactions demonstrate the intricate crosstalk between different biological pathways and processes mediated by both PDEGs and PDEG-interacting genes, which is critical for the establishment of drug response networks. Over-expressing or suppressing the gene expression levels of either PDEGs or PDEG-interacting genes will therefore “skew” the activities of these networks and alter drug responses.

Our study indicates that perturbing the activities of PDEG-interacting genes in drug response networks indeed can lead to altered drug sensitivity. However, only approximately 50% of tested PDEG-interacting genes show predicted change of drug response directionality (e.g. become more sensitive or resistant) by phenotypic frequency (PF) score upon RNAi knockdown. We reason that the key reason for this discrepancy is the lack of annotations for the nature of protein-protein interactions in current human PPI network used in this work. In other words, protein-protein interactions are marked with “signs”, i.e. activation, inhibitory, and neutral interactions. Activation interactions lead to functional activation of targeted genes, whereas inhibitory interactions produce the opposite effect. Neutral interactions do not directly cause activation or inhibition of target genes but are essential for maintaining the functionality of multi-component protein complexes such as interactions of ribosomal protein subunits in ribosome.

**Figure 5:**
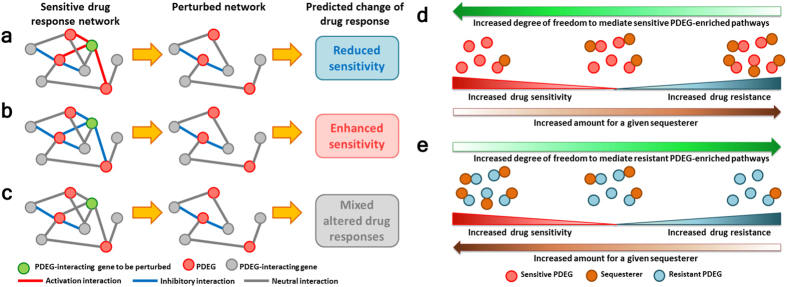
Plausible molecular mechanisms that regulate directionality of drug response when a PDEG-interacting gene is perturbed. (**a**) Reduced sensitivity to drug is predicted if the expression of a PDEG-interacting gene that exhibits positive (activation) interactions (indicated by red lines) to multiple PDEGs is suppressed. (**b**) Enhanced sensitivity to drug is predicted if the expression of a PDEG-interacting gene that exhibits negative (inhibitory) interactions (indicated by blue lines) to multiple PDEGs is suppressed. (**c**) Mixed altered drug responses (enhanced or reduced drug sensitivity or non-response) is predicted if the expression of a PDEG-interacting gene that exhibits neutral interactions to multiple PDEGs is suppressed. (**d**) Effects of a single PDEG-interacting gene from sensitive response network in modulating drug sensitivity. Increased levels of a PDEG-interacting gene sequester its interacting PDEGs and restrict them from interacting with other PDEGs or PDEG-interacting genes, thereby promoting drug resistance. However, decreasing the level of the PDEG-interacting gene frees its interacting PDEGs to interact with other PDEGs or PDEG-interacting genes and gives rise to enhanced drug sensitivity. (**e**) Effects of a single PDEG-interacting gene from resistant response network in modulating drug sensitivity. Increased levels of a PDEG-interacting gene sequester its interacting PDEGs and restrict them from interacting with other PDEGs or PDEG-interacting genes, thereby promoting drug sensitivity. However, decreasing the level of the PDEG-interacting gene frees its interacting PDEGs to interact with other PDEGs or PDEG-interacting genes and gives rise to enhanced drug resistance.

Here, we provide two plausible scenarios to explain our observed results. The first scenario is the “sign influence” of protein-protein interactions (Fig. 5a–c), and the second scenario is the “sequesterer hypothesis”, which indicates the consequences of interfering with the levels of interacting partners for a given protein complex (Fig. 5d,e). Using a sensitive drug response network (DRN) as an illustrative example, and with the underlying assumption that the activities of PDEGs are involved in conferring drug sensitivity, RNAi knockdown of a PDEG-interacting gene that exhibits activation interactions with multiple PDEGs will result in reduced drug sensitivity (Fig. 5a). The opposite results will be obtained if a PDEG-interacting gene shows inhibitory interactions with multiple PDEGs (Fig. 5b). However, if the interactions of a PDEG-interacting gene with multiple PDEGs are neutral, mixed altered drug responses such as enhanced sensitivity, reduced sensitivity, or non-responsiveness might be observed (Fig. 5c), depending on the functional roles of the protein complexes in the drug response phenotypes.

We propose the “sequesterer hypothesis” to illustrate the possible consequences of perturbing PDEG-interacting genes that show neutral interactions with PDEGs as predicted by PF score. The underlying assumption is PDEGs are essential in mediating drug response phenotypes and that formation of protein complexes between PDEG and PDEG-interacting genes can switch the roles of PDEGs in drug response networks. In other words, PDEG-interacting genes may act as “sequesterers” in modulating drug response phenotypes (Fig. 5d,e). As illustrated in Fig. 5d, over-expressing a particular PDEG-interacting gene in a sensitive response network will lead to the sequestering of its corresponding PDEGs, thus preventing them from interacting with other PDEG-interacting genes involved in pathways that confer drug sensitivity and, as a result, promote drug resistance. On the other hand, suppressing the expression of a given PDEG-interacting gene allows its corresponding PDEGs to have a higher degree of freedom to interact with other interacting partners to confer drug sensitivity and, therefore, enhance sensitivity to a drug. In contrast, as illustrated in Fig. 5e, over-expressing a particular PDEG-interacting gene in a resistant network results in the sequestering of its corresponding PDEGs to interact with other genes that confer drug resistance, thereby disrupting the drug resistant phenotype. In contrast, suppressing the expression of a given PDEG-interacting gene promotes drug resistance due to the higher degree of freedom for PDEGs to interact with other PDEG-interacting genes to mediate a drug resistant phenotype and, therefore, to promote drug resistance.

Our results therefore implicate mixed mechanisms that involve the influences of signed protein interactions and sequesterer effects in modulating the properties of drug response networks. The human PPI network used in this work does not contain sign information for protein-protein interactions, thus hampering a more conclusive understanding of how signed protein interactions affect drug response phenotype. Future studies incorporating resources such as RNAi phenotypic screening in order to reconstruct a signed human PPI network using an approach developed by Vinayagam *et al*.^44^ are warranted.

Another key issue in network analyses is that hub genes tend to be false positives due to their high connectivity in PPI network. In this work, PDEGs are defined based on their upregulation in a specific drug response phenotype and are not necessarily hubs in the PPI network. If they are indeed hubs, we anticipate these hub PDEGs would play an important role in a drug response phenotype. In addition, the incorporation of frequency to compute PF scores for N1 and N2 PDEG-interacting genes across M gene pairs can to a certain extent reduce false positive due to hubs because the genes in the M gene pair are essentially determined by the identity of PDEGs. However, we do find hub genes in our study, e.g. UBC (ubiquitin C) and HSP90AA1 (a heat shock protein). Our experimental results indicate UBC is strongly consistent with predicted response directionality in anthracycline (Table 1), but not consistent with response directionality in taxane (Table 2). However, perturbing UBC indeed altered sensitivity to both anthracyclines and taxanes. Perturbing the HSP90AA1 gene showed strong consistency with the predicted response directionality for taxane (Table 2) but non-responsive to anthracycline (Table 1). Our results therefore indicate that we should be careful in interpreting the role of hub PDEG-interacting genes because the current method cannot fully eliminate false positives due to the high network connectivity of hubs. However, researchers are encouraged to experimentally test whether these hub PDEG-interacting genes alter drug sensitivity. If upon genetic perturbation they indeed affect drug sensitivity, these key modulators of drug response phenotypes should be included in the design of systems-based therapeutic prescriptions.

The current study highlights a new perspective for designing therapeutics in the field of systems pharmacology. The conventional systems pharmacology approach is to identify combinations of drugs with multiple therapeutic targets to achieve synergistic effects^45^,^46^; however, our work reveals that while designing drugs to target specific therapeutic candidates is important, it is also important to modulate the activities of DRNs. Our work therefore indicates that two types of ingredients are needed for the development of systems-based therapeutics: therapeutics that mainly exert therapeutic effects by targeting to disease culprits, and drug response phenotypic modulators that serve to intervene in the activities of drug response networks to prevent or re-sensitize drug resistance.

Using basal transcriptomics data (i.e. before drug is administered), P-Map provides an alternative network-based strategy to dissect drug response networks for a given drug and identify potential gene candidates whose activities, when manipulated, can alter the properties of drug response network. This opens a new avenue to discover chemical modulators that can interfere with the activities of selected gene candidates to modulate drug response networks with the ultimate goal of maximizing efficacy and minimizing resistance to administered drugs. In other words, drug discovery is not limited to identifying chemicals that target to disease culprits but can be extended to a broader scope in finding “modulators” for drug response networks.

## Materials and Methods

### Genome-wide mRNA Expression Data

Whole Genome expression data for cell lines was obtained with Affymetrix U133 plus 2.0 expression array chip. The RNA extraction and the expression array assays were performed following the Affymetrix GeneChip® expression technical manual (Affymetrix, Inc., Santa Clara, CA). Before the assay, RNA quality was tested using an Agilent 2100 Bioanalyzer. The Affymetrix GeneChip® contains over 54,000 probe sets whose design is based on build 34 of the Human Genome Project. The mRNA expression array data were normalized on the log2 scale using GCRMA methodology^47^. The mRNA expression data for the entire set of LCL cell lines has been assigned GEO accession number GSE23120.

### LCLs and Cytotoxicity Assays

The LCL cell line samples were purchased from the Coriell Cell Repository (Camden, NJ) and collected and anonymized by the National Institute of General Medical Sciences (NIGMS). Cells were plated at a density of 5 × 10^4^ cells per well in triplicate in 96-well plates (Corning, NY). One hour after plating, cells were treated with epirubicin, doxorubicin, paclitaxel or docetaxel. The CellTiter96 Aqueous Non-Radioactive Cell Proliferation Assays (Promega, Madison, WI) were performed as described by the manufacturer after 72 h incubations. Plates were read in a Safire2 microplate reader (Tecan AG, Switzerland), with subsequent cytotoxicity measurements recorded at various doses of epirubicin, doxorubicin, paclitaxel and docetaxel for the cell lines. To determine EC50 values for each drug, a logistic function was fitted to the cytotoxicity data using the R package drc (http://cran.r-project.org/). Cell IDs and corresponding EC50 values used in this study are provided in Supplementary Data 1.

### The P-Map algorithm

Samples were ranked by EC50 value in respect to each LCL cell line to a given drug (Supplementary Data 1) and defined as a ‘sensitive’ or ‘resistant’ phenotype by taking 15 samples with the lowest EC50 values to define a sensitive phenotype and 15 samples with the highest EC50 values to define a resistant phenotype (Supplementary Figure 1). This step was performed for each ethnic group and drug and resulting in a total of 24 drug response conditions defined by ethnic groups and use of drugs. We used a template matching approach^27^ to define differentially expressed genes as sensitive or resistant phenotypes for each ethnic group and drug. Phenotypic differentially expressed genes (PDEGs) should show up-regulation in at least two cell lines grouped under the same drug response phenotype (e.g. anthracycline sensitive). For instance, PDEGs for the anthracycline sensitive phenotype are up-regulated in no less than two sensitive cell lines in respect to doxorubicin or epirubicin within three ethnic groups (African, Caucasian, and Chinese).

Next, the respective PDEGs were mapped to a protein-protein interaction (PPI) network that was built from the iRefIndex version 14.0^48^. Here, we aim to uncover interactions that are potentially important in mediating drug response phenotypes in an unbiased, assumption-free but data-oriented manner. Available interactions from iRefIndex are therefore included in the PPI network used in this work. These interactions include direct interaction, physical association, colocalization, association, covalent binding, methylation reaction, phosphorylation reaction, cleavage reaction, genetic interaction, dephosphorylation reaction, ubiquitination reaction, hydroxylation reaction, self-interaction, protein cleavage, acetylation reaction, deubiquitination reaction, ADP ribosylation reaction, deacetylation reaction, enzymatic reaction, palmitoylation reaction, sumoylation reaction, disulfide bond, RNA cleavage, DNA strand elongation, oxidoreductase activity electron, transfer reaction gtpase, reaction, neddylation reaction, transglutamination reaction, demethylation reaction, isomerase reaction, proline isomerization reaction and phosphotransfer reaction. We removed self-loops and multiple edges creating a final iRefIndex index network containing 15 608 proteins and 180 044 interactions.

Interacting with at least two PDEGs is the minimal criterion for PDEG-interacting genes to maximize PDEG inclusion and to preserve functionally relevant genes that interact with PDEGs in the network. To maximize the inclusion of PDEGs in a drug response network we define two types of genes based on their interactions with PDEGs and topological properties in the PPI network. Genes that act as first-tier interacting partners (direct interaction) to at least two PDEGs were termed N1 PDEG-interacting genes. Genes that directly interact with N1 genes but at the same time also interact with at least two PDEGs were termed N2 PDEG-interacting genes. Therefore, N2 genes always interact with PDEGs and N1 genes, but N1 genes do not necessarily interact with N2 genes. Interactions among PDEGs, N1, and N2 genes formed DRN in respect to a drug response phenotype, such as anthracycline resistant phenotype. The computation step is as follows: For each PDEG, find its first interacting partners in the PPI network. If the overlap between these genes and PDEGs is larger than two, they are called the N1 PDEG-interacting genes. We then find the first interacting partners of each N1 gene. If the overlap between these genes and PDEGs is larger than 2 (excluding ubiquitin C (UBC)), they are called the N2 PDEG-interacting genes. We then computed the frequencies in which genes in N1 and N2 appear across all gene pairs PDEG-N1. Essentially, this step generated a matrix of MxF dimensions where M is the number of gene pairs PDEG-N1 whose first interacting partners fulfill the filtering steps above and F = 2 refers to the N1 and N2 gene lists, respectively. Then, we computed how many times genes in N1 and N2 appear across M gene pairs to find their frequency of occurrence as interacting partners of PDEGs. The frequency was used to rank gene candidates. The lists for N1 and N2 genes and their respective computed frequencies for each drug response phenotype are given in Supplementary Data 4.

### Pathway enrichment analyses for PDEGs

Pathway analyses for PDEGs associated with phenotypes sensitive or resistant to anthracyclines and taxanes were performed with a web-based engine at WebGestalt (**WEB**-based **GE**ne **S**e**T A**na**L**ysis **T**oolkit) at http://bioinfo.vanderbilt.edu/webgestalt/ using gene symbols mapped to human genome. Canonical pathways from a KEGG database were queried. A Hypergeometric test together with a Bonferroni test for multiple hypothesis testing corrections was performed. Pathways (minimum 5 genes) with adjusted p-value < 0.05 were considered significantly enriched.

### Gene set enrichment analyses for N1 PDEG-interacting genes

The relative occurrence of a given gene as a PDEG-interacting gene across M PDEG gene pairs in relative to sensitive and resistant network is estimated with its phenotypic frequency (PF) score. For N1 PDEG-interacting genes is computed as follows: PF Score = frequency_sensitive_ N1_i_ − frequency_resistant_ N1_i_, where frequency corresponding to how many times a given N1 gene appears across a list of M gene pairs (PDEG-N1 gene pairs). The PF score therefore gives a rough estimate of the relative occurrence of given N1 PDEG-interacting genes in a given phenotype. A positive PF score indicates higher occurrence for a given N1 PDEG-interacting gene in a sensitive network and it is assumed to play a role in modulating the properties of a sensitive network. The same reasoning is applied for a negative PF score for a N1 PDEG-interacting gene in a resistant network. We next ranked N1 PDEG-interacting genes based on their PF scores and the pre-ranked option of GSEA^49^ was performed using canonical pathway (CP) gene signatures. The enrichments were performed by randomly swapping the gene labels for 1000 permutations. Pathways with nominal p-value (NP) < 0.05 were deemed statistically significant.

### Human triple negative breast cancer cell lines, drugs and cytotoxicity assays

Human triple negative breast cancer MDA-MB231 and BT549 cell lines were obtained from the American Type Culture Collection (ATCC) (Manassas, VA). MDA-MB231 cells were cultured in L15 supplemented with 10% FBS (Mediatech), and BT549 cells were maintained in RPMI medium 1640 supplemented with 10% FBS (Mediatech). Epirubicin, doxorubicin, paclitaxel and docetaxel were purchased from Sigma-Aldrich (Milwaukee, WI). Drugs were dissolved in DMSO and aliquots of stock solutions were frozen at −80 °C. Cell proliferation assays were performed in triplicate at each drug concentration. Cytotoxicity assays with the lymphoblastoid and tumor cell lines were performed in triplicate at each dose. Specifically, 90 μL of cells (5 × 10^3^ cells/mL) were plated into 96-well plates (Corning, NY) and were treated with 10 μl of epirubicin or doxorubicin at final concentrations of 0, 0.0156, 0.03125, 0.0625, 0.125, 0.25, 0.55, 1, and 2 μM. Similarly, cells were treated with paclitaxel or docetaxel at 0, 0.01, 0.1, 1, 10, 50, 100, 1000, and 5000 nM. After incubation for 72 hours, 20 μL of CellTiter 96® AQueous Non-Radioactive Cell Proliferation Assay solution (Promega Corporation, Madison, WI) was added to each well. Plates were read in a Safire2 plate reader (Tecan AG, Switzerland).

### Transient transfection and RNA interference

Specific siGENOME siRNA SMARTpool® reagents against a given gene, as well as a negative control, siGENOME Non-Targeting siRNA Pool #2, were purchased from Dharmacon Inc. (Lafayette, CO). Human triple negative breast cancer MDA-MB231 and BT549 cell lines were used to perform the siRNA knockdown studies. The lipofectamine RNAiMAX transfection reagent (Invitrogen, Carlsbad, CA) was used for siRNA reverse or forward transfection. Specifically, cells were seeded into 96-well plates and were mixed with siRNA-complex consisting of 20–50 nM of specific siGENOME siRNA SMARTpool or non-targeting negative control (Dharmacon) and the lipofectamine RNAiMAX transfection reagent. Significance of the IC50 values between negative control siRNA and gene-specific siRNA was determined by a two-tailed unpaired t-test with p-value < 0.05 is considered statistically significant.

## Additional Information

**How to cite this article**: Cairns, J. *et al*. A network-based phenotype mapping approach to identify genes that modulate drug response phenotypes. *Sci. Rep.* **6**, 37003; doi: 10.1038/srep37003 (2016).

**Publisher’s note:** Springer Nature remains neutral with regard to jurisdictional claims in published maps and institutional affiliations.

## Supplementary Material

Supplementary Information

Supplementary Information

Supplementary Information

Supplementary Information

Supplementary Information

Supplementary Information

## Figures and Tables

**Table 1:** Summary of RNAi knockdown experiments on selected PDEG-interacting genes from anthracycline (doxorubicin and epirubicin) response network in two triple-negative breast cancer (TNBC) cell lines (BT549 and MDA-MB-231).

PDEG-Interacting Gene	Change of Anthracycline Sensitivity in BT549 Cell Line	Change of Anthracycline Sensitivity in MDA-MB-231 Cell Line	Sum N1 + N2 PF Score	Consistency to sign of PF score
NOTCH2	Non-responsive	↓ Sensitivity	−21	Moderate consistency to MDA-MB-231 cell line
CAV1	Non-responsive	↓ Sensitivity	−331	Strong consistency to MDA-MB-231 cell line
BCL2L11	Non-responsive	↓ Sensitivity	−238	Strong consistency to MDA-MB-231 cell line
CXCL9	Non-responsive	↓ Sensitivity to epirubicin, non-responsive to doxorubicin	−8	Weak consistency to MDA-MB-231 cell line
AKAP7	Non-responsive	Non-responsive	−2	Inconsistent
IRF5	↓ Sensitivity	↓ Sensitivity	−32	Moderate Consistency
CD244	↓ Sensitivity	↓ Sensitivity	−1	Weak Consistency
LAPTM4B	↓ Sensitivity	↓ Sensitivity	−46	Moderate Consistency
MXRA7	↓ Sensitivity	↓ Sensitivity	−2	Weak Consistency
GNA15	↓ Sensitivity	↓ Sensitivity	−204	Strong Consistency
RHOBTB3	↓ Sensitivity	↓ Sensitivity	−1	Weak Consistency
UBC	↓ Sensitivity	↓ Sensitivity	−1101	Strong Consistency
EP300	Non-responsive	Non-responsive	−308	Inconsistent
ELAVL1	Non-responsive	↓ Sensitivity to epirubicin, non-responsive to doxorubicin	−73	Moderate consistency to MDA-MB-231 cell line
HDAC2	Non-responsive	Non-responsive	−146	Inconsistent
HSP90AA1	Non-responsive	Non-responsive	−756	Inconsistent
GRB2	↓ Sensitivity	↓ Sensitivity	−495	Strong Consistency
ZAP70	↓ Sensitivity	↓ Sensitivity	−322	Strong Consistency
FOXP1	↓ Sensitivity	↓ Sensitivity	−2	Weak Consistency

Response indications refer to both types of anthracyclines (doxorubicin and epirubicin) and two TNBC cell lines unless otherwise indicated. PDEG: Phenotypic differentially expressed genes; PF: phenotypic frequency; non-response: no change to anthracycline sensitivity. Since a negative PF score is an estimated measure of the number of times a gene is found in a resistant network, the consistency of a negative PF score with observed reduced drug sensitivity are qualitatively divided into three categories: weak consistency, if the PF score absolute value < 10; moderate consistency, if the PF score absolute value is between 10 and 100; strong consistency, if the PF score absolute value > 100.

**Table 2:** Summary of RNAi knockdown experiments on selected PDEG-interacting genes from taxane (docetaxel and paclitaxel) response network in two triple-negative breast cancer (TNBC) cell lines (BT549 and MDA-MB-231).

PDEG-Interacting Gene	Change of Taxane Sensitivity in BT549 Cell Line	Change of Taxane Sensitivity in MDA-MB-231 Cell Line	Sum N1 + N2 PF Score	Consistency to sign of PF score
FOXP1	Non-responsive	Non-responsive	−48	Inconsistent
NME7	↑ Sensitivity	↑ Sensitivity	−41	Inconsistent
EPS8	↑ Sensitivity	↑ Sensitivity	−87	Inconsistent
IL1R1	↑ Sensitivity	↑ Sensitivity	−9	Inconsistent
DDX17	↑ Sensitivity	↑ Sensitivity	−7	Inconsistent
CNR1	↑ Sensitivity	↑ Sensitivity	−2	Inconsistent
UBC	↓ Sensitivity	↓ Sensitivity	274	Inconsistent
ELAVL1	↑ Sensitivity	↑ Sensitivity	7	Weak Consistency
HMOX1	↑ Sensitivity	↑ Sensitivity	17	Moderate Consistency
HCK	↑ Sensitivity	↑ Sensitivity	50	Moderate Consistency
TXN	↑ Sensitivity	↑ Sensitivity	1	Weak Consistency
HSP90AA1	↑ Sensitivity	↑ Sensitivity	138	Strong Consistency
NOTCH2	↑ Sensitivity	↑ Sensitivity	3	Weak Consistency

The indications are the same as Table 1.
