# Supplementary material for: A network-based phenotype mapping approach to identify genes that modulate drug response phenotypes
Source: Sci Rep. 2016 Nov 14;6:37003. doi: 10.1038/srep37003 (PMC5107984; doi:10.1038/srep37003)
Supplement: Supplementary Information [file srep37003-s1.pdf]

A network-based phenotype mapping approach to identify genes that modulate drug response phenotypes

Authors: Junmei Cairns[1,]†, Choong Yong Ung[1,]†, Edroaldo Lummertz da Rocha[1,]†, Cheng Zhang[1], Cristina Correia[1], Richard Weinshilboum[1], Liewei Wang[1,]* and Hu Li[1,]*

Affiliation: [1]Department of Molecular Pharmacology and Experimental Therapeutics, Mayo Clinic College of Medicine, Rochester, MN 55905, USA.

†These authors contributed equally to this work.

***Corresponding author:**

Hu Li, Ph.D.
Department of Molecular Pharmacology and Experimental Therapeutics
Mayo Clinic College of Medicine
Gonda Building, 19-408
200 First Street SW
Rochester, MN 55905
Office: 1-507-293-1182
Fax: 1- 507-284-4455
E-mail: li.hu@mayo.edu

Liewei Wang, M.D. Ph.D.
Department of Molecular Pharmacology and Experimental Therapeutics
Mayo Clinic College of Medicine
Gonda Building, 19-460
200 First Street SW
Rochester, MN 55905
Office: 1-507- 774-5264
Fax: 1- 507-284-4455
E-mail: Wang.Liewei@mayo.edu

Supplementary Figure Legends

Supplementary Figure 1. **Plots for distribution of EC50 values for LCL cell lines of African, Caucasian, and Chinese origin in response to anthracyclines (doxorubicin and epirubicin) and taxanes (docetaxel and paclitaxel).** The 15 cell lines that are defined as sensitive (lower end of plots) and resistant (upper end) indicated in red and used for subsequent analyses.

Supplementary Figure 2. Number of overlapped PDEGs between LCL-derived taxane sensitive and resistant phenotypes with paclitaxel sensitive and resistant phenotypes for breast cancer cell lines from Cancer Cell Line Encyclopedia (CCLE).

Supplementary Figure 3. **PDEG- and PDEG-interacting gene-enriched biological pathways in DRNs that confer taxane sensitivity and resistance**. (**A**) Enriched pathways for PDEGs in taxane sensitive response network; (**B**) Enriched pathways for PDEGs in taxane resistant response network; (**C**) Enriched pathways in Gene Set Enrichment Analysis (GSEA) using pre-ranked option for PDEG-interacting genes in taxane sensitive and resistant response networks, ranked with phenotypic frequency (PF) scores. Red: enriched pathways in taxane sensitive network (positive PF scores); blue: enriched pathways in taxane resistant network (negative PF score). Four selected GSEA-enriched pathways for PDEG-interacting genes in taxane sensitive network are indicated at right panel.

Supplementary Figure 4. **Response curves of MTS assays before and after knockdown of selected PDEG-interacting genes in BT549 and MDA-MB-231 triple-negative breast cancer**

(TNBC) cell lines treated with taxanes (docetaxel and paclitaxel). (**A**) siRNA knockdown followed by docetaxel treatment in BT549 cells; (**B**) siRNA knockdown followed by paclitaxel treatment in BT549 cells; (**C**) siRNA knockdown followed by docetaxel treatment in MDA-MB-231 cells; (**D**) siRNA knockdown followed by paclitaxel treatment in MDA-MB-231 cells. Red: candidate siRNA; blue: negative control siRNA. X-axis: drug concentrations in logarithm scale of nM; Y-axis: fraction of surviving cells.

Supplementary Figure 5. **Network for taxane-sensitive N1 PDEG-interacting genes**. A portion of the taxane-sensitive drug response network showing N1 PDEG-interacting genes (green nodes) exhibiting high PF scores and their corresponding interacting PDEGs (red nodes).

Supplementary Figure 6. **Computed distributions for the degree of connectedness for anthracycline resistant and taxane sensitive networks**.

SUPPLEMENTARY DATA

Supplementary Data 1. **Cytotoxicity data for corresponding cell lines.**

Supplementary Data 2. **Heatmaps of upregulated genes in all 24 drug response conditions**.

Supplementary Data 3. **Lists of PDEGs and the number of their occurrences with respect to the same drug response phenotype**. The nominal p-value for a gene as PDEG is defined as the probability (approximated by the frequency from permutations via permuting EC50 labels) of picking this gene by chance.

Supplementary Data 4. **PDEG-interacting genes and their respective computed frequencies and PF scores in both anthracycline and taxane response networks**. The nominal p-value for a gene as N1 for sensitive is defined as the frequency of picking this gene as N1 sensitive and having a larger frequency_sensitive by chance. The same criterion also applied to N1 for resistant, and for N2 sensitive, and N2 resistant genes. Since each gene has both frequency_sensitive and frequency_resistant, the smaller nominal p-value of the 2 should be used to describe the significance. The nominal p-value (1000 permutations for EC50 labels) for a gene as N1 or N2 is defined for sensitive and resistant separately, e.g. nominal p-value for an N1 gene as related to sensitive:

Nominal-P = Number of (frequency_sensitive as N1 scores from permutation >= frequency_sensitive as N1 scores in the results) / number of permutations.

Supplementary Data 5. Results for statistical tests for PDEGs enriched pathways. The nominal p-value for a KEGG pathway is defined as finding this pathway as significant (adjusted pvalue>=0.2) by chance. Over-representation test was performed for PDEGs in each permutation (total 1000 permutations for EC50 labels) and the nominal p-values for the KEGG pathways are defined as:

Number of finding a pathway as significant / number of permutations.

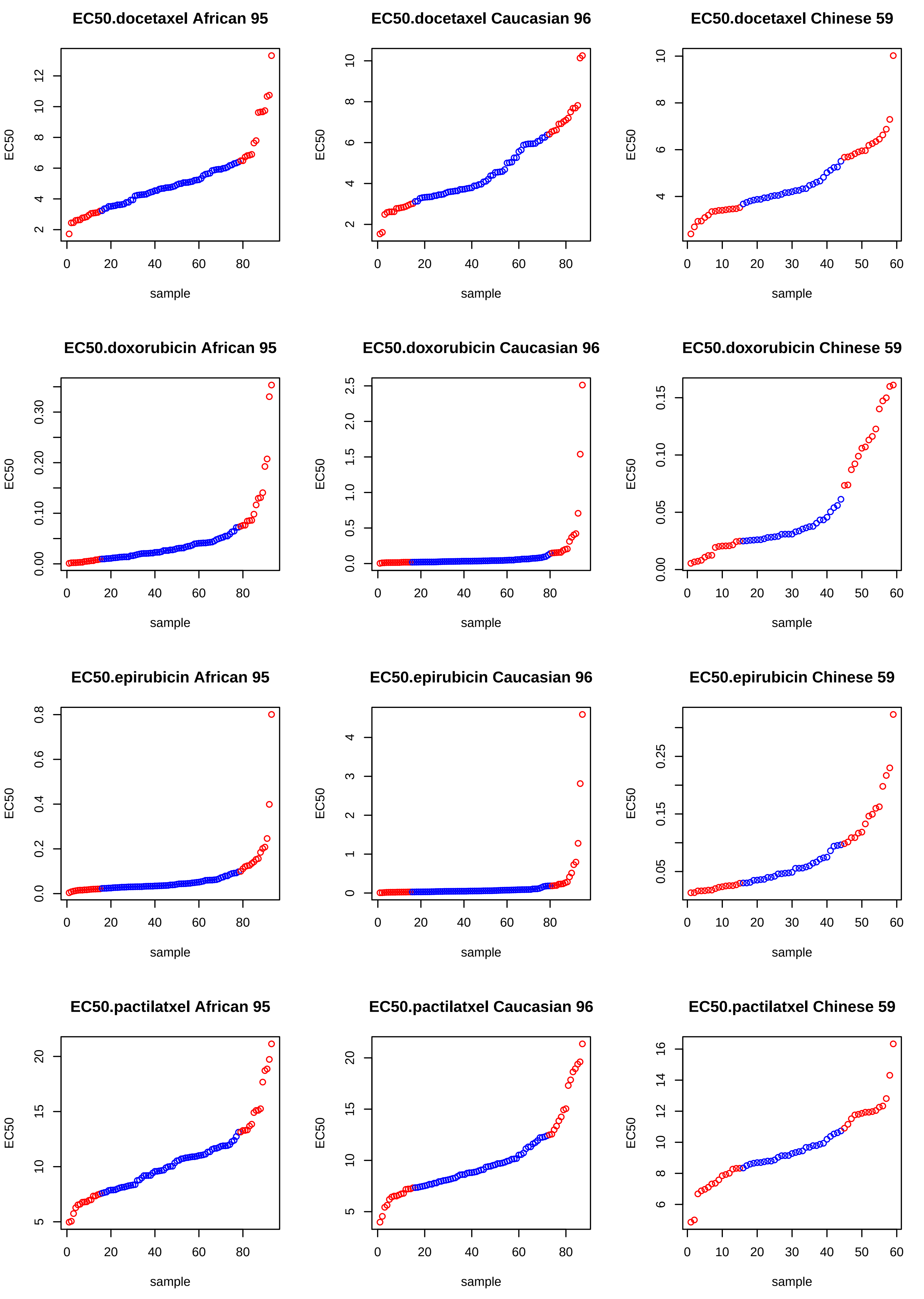
EC50.docetaxel African 95
EC50.docetaxel Caucasian 96
EC50.docetaxel Chinese 59
EC50.doxorubicin African 95
EC50.doxorubicin Caucasian 96
EC50.doxorubicin Chinese 59
EC50.epirubicin African 95
EC50.epirubicin Caucasian 96
EC50.epirubicin Chinese 59
EC50.pactilatxel African 95
EC50.pactilatxel Caucasian 96
EC50.pactilatxel Chinese 59
EC50
sample

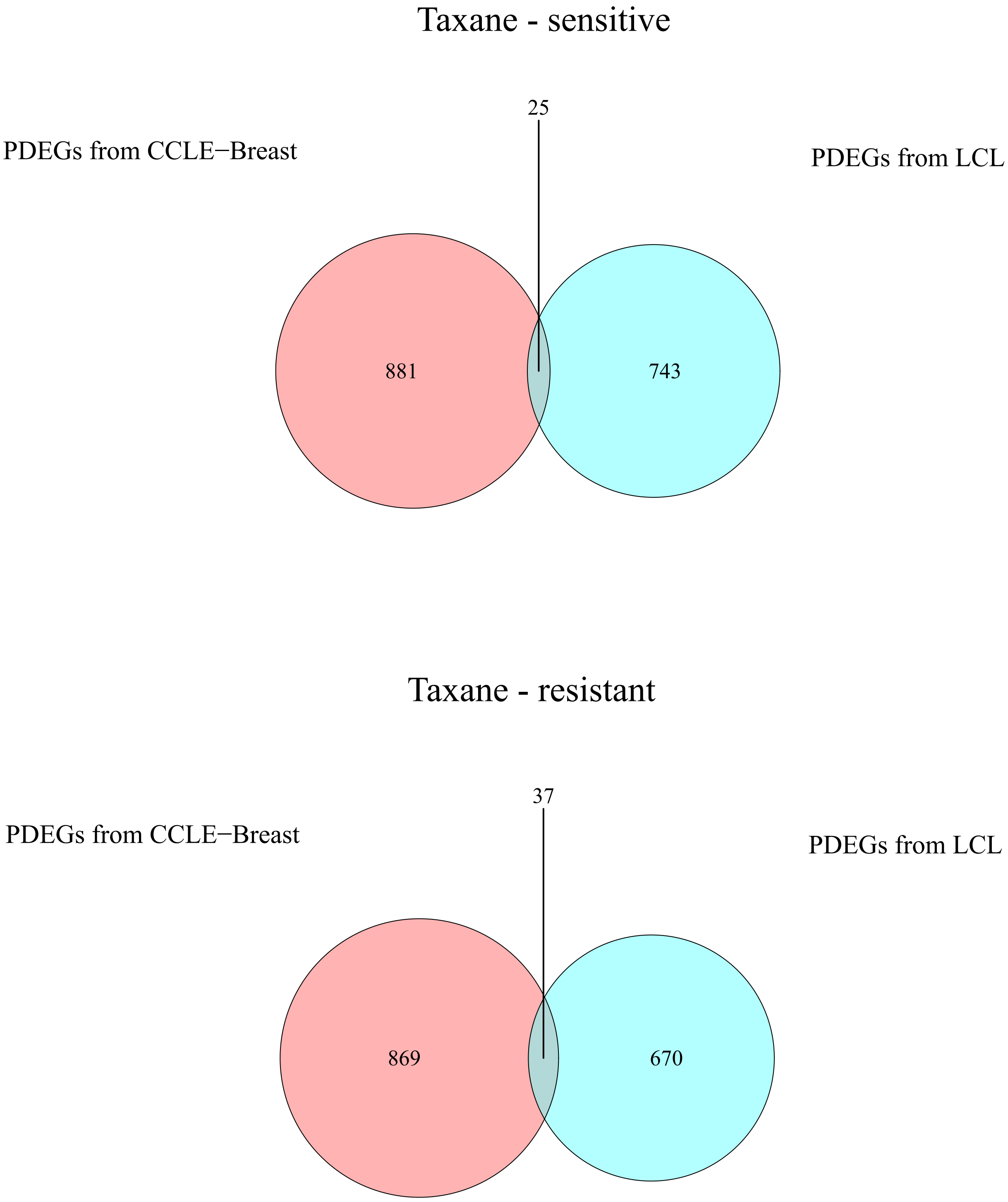
Taxane - sensitive
PDEGs from CCLE−Breast
PDEGs from LCL
25
881
743
Taxane - resistant
PDEGs from CCLE−Breast
PDEGs from LCL
37
869
670

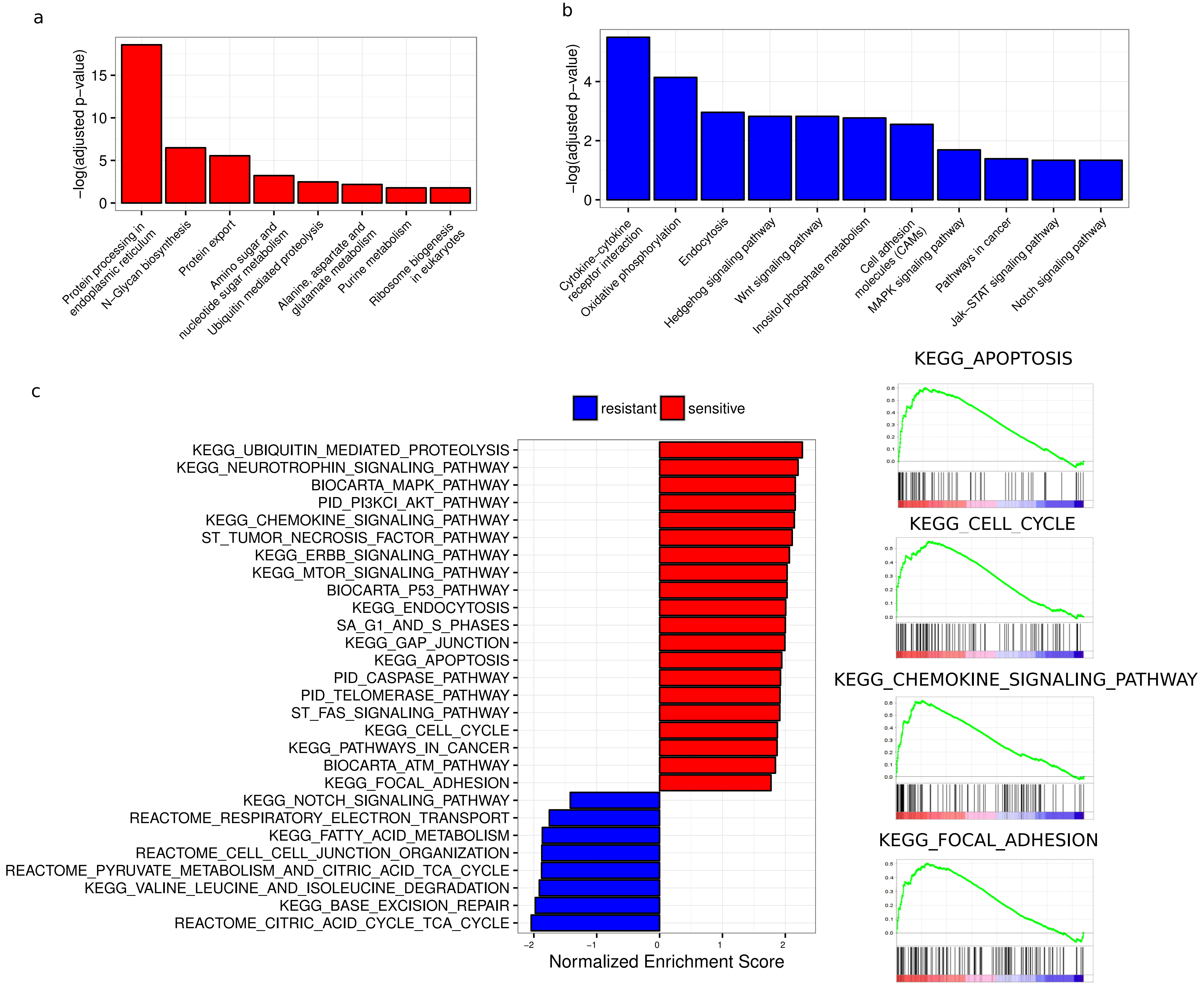
a
−log(adjusted p−value)
0
5
10
15
Protein processing in endoplasmic reticulum
N−Glycan biosynthesis
Protein export
Amino sugar and nucleotide sugar metabolism
Ubiquitin mediated proteolysis
Alanine, aspartate and glutamate metabolism
Purine metabolism
Ribosome biogenesis in eukaryotes
b
−log(adjusted p−value)
0
2
4
Cytokine−cytokine receptor interaction
Oxidative phosphorylation
Endocytosis
Hedgehog signaling pathway
Wnt signaling pathway
Inositol phosphate metabolism
Cell adhesion molecules (CAMs)
MAPK signaling pathway
Pathways in cancer
Jak−STAT signaling pathway
Notch signaling pathway
c
resistant
sensitive
KEGG_UBIQUITIN_MEDIATED_PROTEOLYSIS
KEGG_NEUROTROPHIN_SIGNALING_PATHWAY
BIOCARTA_MAPK_PATHWAY
PID_PI3KCI_AKT_PATHWAY
KEGG_CHEMOKINE_SIGNALING_PATHWAY
ST_TUMOR_NECROSIS_FACTOR_PATHWAY
KEGG_ERBB_SIGNALING_PATHWAY
KEGG_MTOR_SIGNALING_PATHWAY
BIOCARTA_P53_PATHWAY
KEGG_ENDOCYTOSIS
SA_G1_AND_S_PHASES
KEGG_GAP_JUNCTION
KEGG_APOPTOSIS
PID_CASPASE_PATHWAY
PID_TELOMERASE_PATHWAY
ST_FAS_SIGNALING_PATHWAY
KEGG_CELL_CYCLE
KEGG_PATHWAYS_IN_CANCER
BIOCARTA_ATM_PATHWAY
KEGG_FOCAL_ADHESION
KEGG_NOTCH_SIGNALING_PATHWAY
REACTOME_RESPIRATORY_ELECTRON_TRANSPORT
KEGG_FATTY_ACID_METABOLISM
REACTOME_CELL_CELL_JUNCTION_ORGANIZATION
REACTOME_PYRUVATE_METABOLISM_AND_CITRIC_ACID_TCA_CYCLE
KEGG_VALINE_LEUCINE_AND_ISOLEUCINE_DEGRADATION
KEGG_BASE_EXCISION_REPAIR
REACTOME_CITRIC_ACID_CYCLE_TCA_CYCLE
−2
−1
0
1
2
Normalized Enrichment Score
KEGG_APOPTOSIS
KEGG_CELL_CYCLE
KEGG_CHEMOKINE_SIGNALING_PATHWAY
KEGG_FOCAL_ADHESION

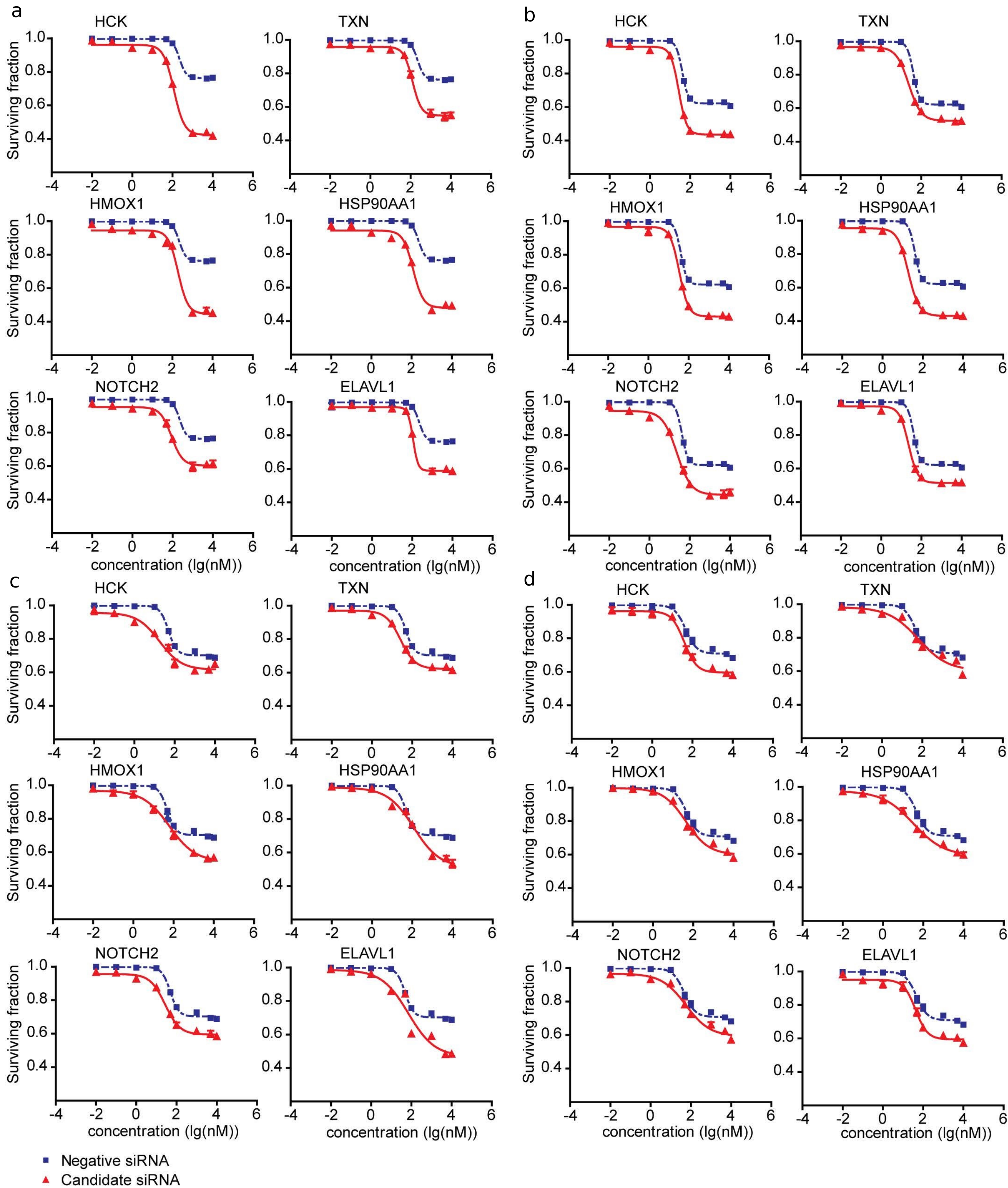
a
b
c
d
HCK
TXN
HMOX1
HSP90AA1
NOTCH2
ELAVL1
Surviving fraction
concentration (lg(nM))
Negative siRNA
Candidate siRNA

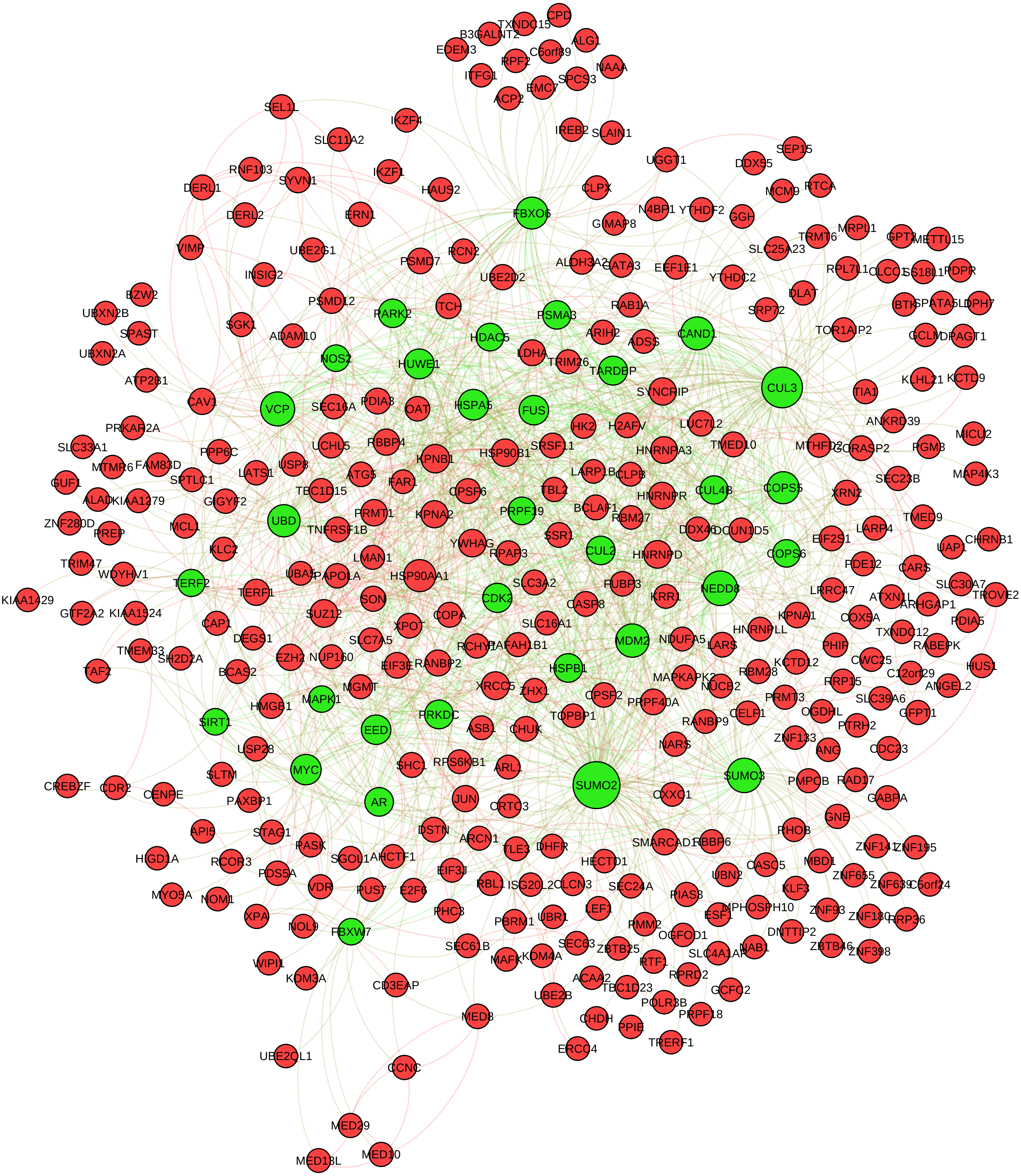
CPD
TXNDC15
B3GALNT2
ALG1
EDEM3
C6orf89
RPF2
NAAA
ITFG1
SPCS3
EMC7
ACP2
SEL1L
IKZF4
IREB2
SLAIN1
SLC11A2
UGGT1
SEP15
DDX55
RNF103
IKZF1
SYVN1
DERL1
HAUS2
CLPX
RTCA
MCM9
FBXO6
N4BP1
YTHDF2
GGH
ERN1
DERL2
GIMAP8
VIMP
MRPL1
GPT2
METTL15
TRMT6
SLC25A23
UBE2G1
RCN2
PSMD7
INSIG2
ALDH3A2
GATA3
EEF1E1
RPL7L1
CLCC1
SS18L1
PDPR
UBE2D2
YTHDC2
BZW2
DLAT
PSMD12
IBTK
SPATA5L1
DPH7
UBXN2B
TCH
PARK2
PSMA3
RAB1A
SRP72
SGK1
HDAC5
ARIH2
CAND1
TOR1AIP2
SPAST
ADAM10
ADSS
GCLM
DPAGT1
UBXN2A
LDHA
NOS2
TRIM26
HUWE1
TARDBP
ATP2B1
KLHL21
KCTD9
CUL3
TIA1
SYNCRIP
CAV1
PDIA3
VCP
SEC16A
HSPA5
OAT
FUS
H2AFV
LUC7L2
ANKRD39
PRKAR2A
HK2
MICU2
TMED10
UCHL5
RBBP4
HSP90B1
SRSF11
HNRNPA3
MTHFD2
CORASP2
PGM3
SLC33A1
PPP6C
KPNB1
MTMR6
FAM83D
USP8
LARP1B
CLPB
MAP4K3
ATG5
LATS1
SEC23B
GUF1
SPTLC1
TBC1D15
FAR1
TBL2
CPSF6
HNRNPR
CUL4B
COPS5
XRN2
ALAD
KIAA1279
GIGYF2
PRKRIP1
BCLAF1
PRPF19
PRMT1
KPNA2
RBM27
TMED9
UBD
ZNF280D
MCL1
TNFRSF1B
DDX46
CCDC124
DCUN1D5
LARP4
CHRNB1
PREP
YWHAG
SSR1
CUL2
EIF2S1
UAP1
RPAP3
HNRNPD
COPS6
KLC2
LMAN1
PDE12
TRIM47
CARS
WDYHV1
UBA5
PAPOLA
HSP90AA1
SLC30A7
TERF2
TERF1
SLC3A2
FUBP3
NEDD8
LRRC47
TROVE2
KIAA1429
SON
CDK2
KRR1
ATXN1L
ARHGAP1
GTF2A2
KIAA1524
SUZ12
COPA
CASP8
KPNA1
COX5A
PDIA5
CAP1
XPOT
SLC16A1
HNRNPLL
TXNDC12
DEGS1
SLC7A5
MDM2
NDUFA5
PHIP
RABEPK
TMEM33
RCHY1
LARS
CWC25
SH2D2A
EZH2
NUP160
PAFAH1B1
KCTD12
HUS1
TAF2
EIF3E
RANBP2
HSPB1
RBM28
C12orf29
BCAS2
MAPKAPK2
RRP15
ANGEL2
MGMT
XRCC5
NUCB2
HMGB1
MAPK1
ZHX1
CPSF2
PRMT3
SLC39A6
PRPF40A
OGDHL
GFPT1
SIRT1
PRKDC
TOPBP1
RANBP9
CELF1
EED
ASB1
CHUK
PTRH2
ZNF133
USP28
NARS
CDC23
ANG
MYC
RPS6KB1
SHC1
ARL1
SLTM
SUMO2
SUMO3
PMPCB
RAD17
CREBZF
CDR2
CENPE
AR
JUN
CXXC1
GABPA
PAXBP1
CRTC3
GNE
API5
STAG1
DSTN
RHOB
ARCN1
SMARCAD1
RBBP6
PASK
TLE3
DHFR
ZNF141
ZNF195
HIGD1A
SGOL1
AHCTF1
HECTD1
CASC5
MBD1
RCOR3
UBN2
PDS5A
EIF3J
ZNF655
ZNF639
C5orf24
VDR
PUS7
E2F6
RBL1
ISG20L2
CLCN3
SEC24A
KLF3
MYO5A
NOM1
PIAS3
MPHOSPH10
ZNF93
ZNF180
RRP36
XPA
PHC3
LEF1
ESF1
NOL9
FBXW7
PBRM1
UBR1
PMM2
OGFOD1
DNTTIP2
SEC61B
SEC63
ZBTB25
SLC4A1AP
NAB1
ZBTB46
ZNF398
WIPI1
MAFK
KDM4A
RTF1
KDM3A
ACAA2
RPRD2
CD3EAP
TBC1D23
GCFC2
UBE2B
MED8
POLR3B
PRPF18
CHDH
PPIE
ERCC4
TRERF1
UBE2QL1
CCNC
MED29
MED10
MED13L

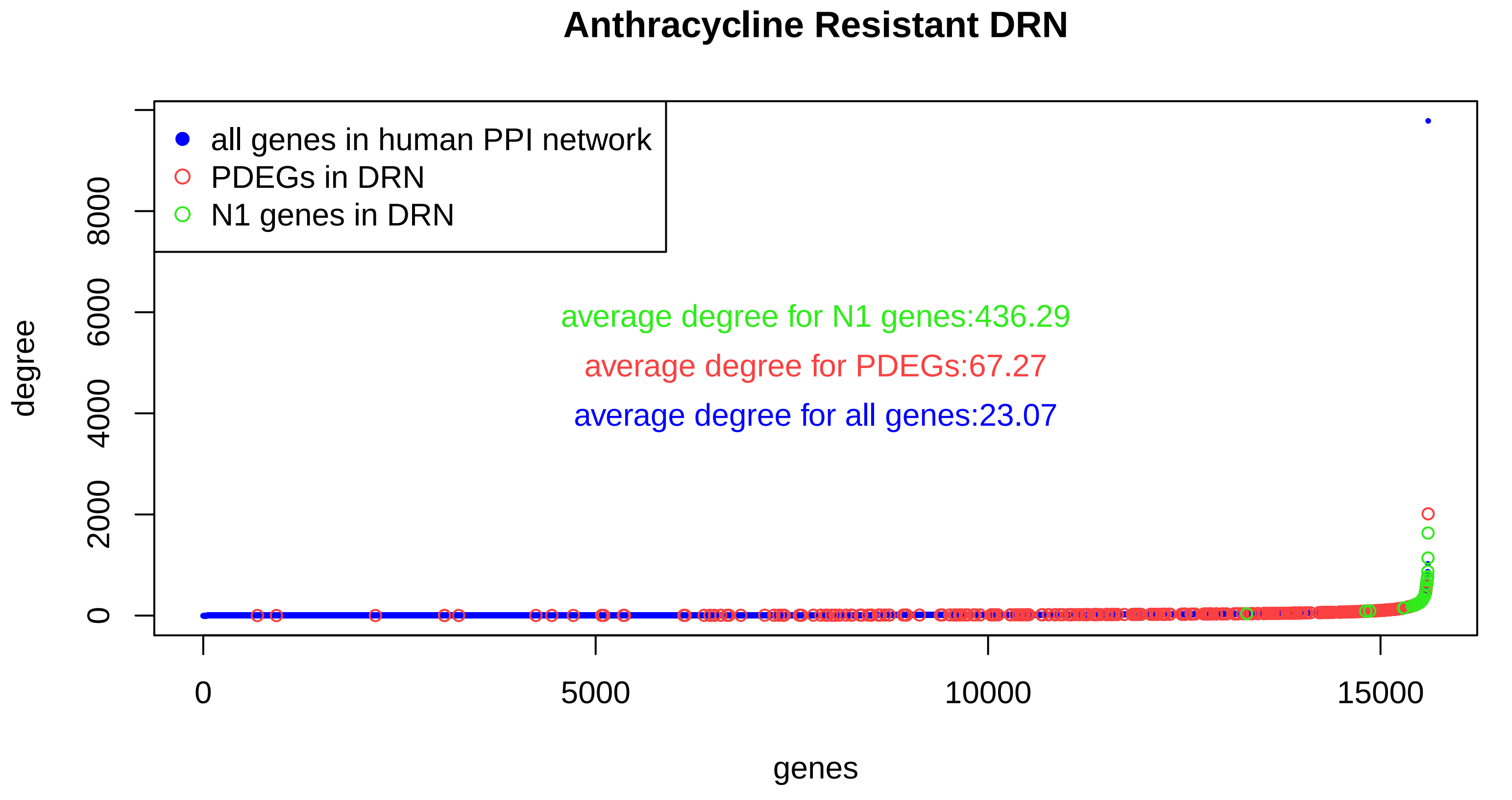

Anthracycline Resistant DRN
all genes in human PPI network
PDEGs in DRN
N1 genes in DRN
average degree for N1 genes:436.29
average degree for PDEGs:67.27
average degree for all genes:23.07
degree
0
2000
4000
6000
8000
0
5000
10000
15000
genes

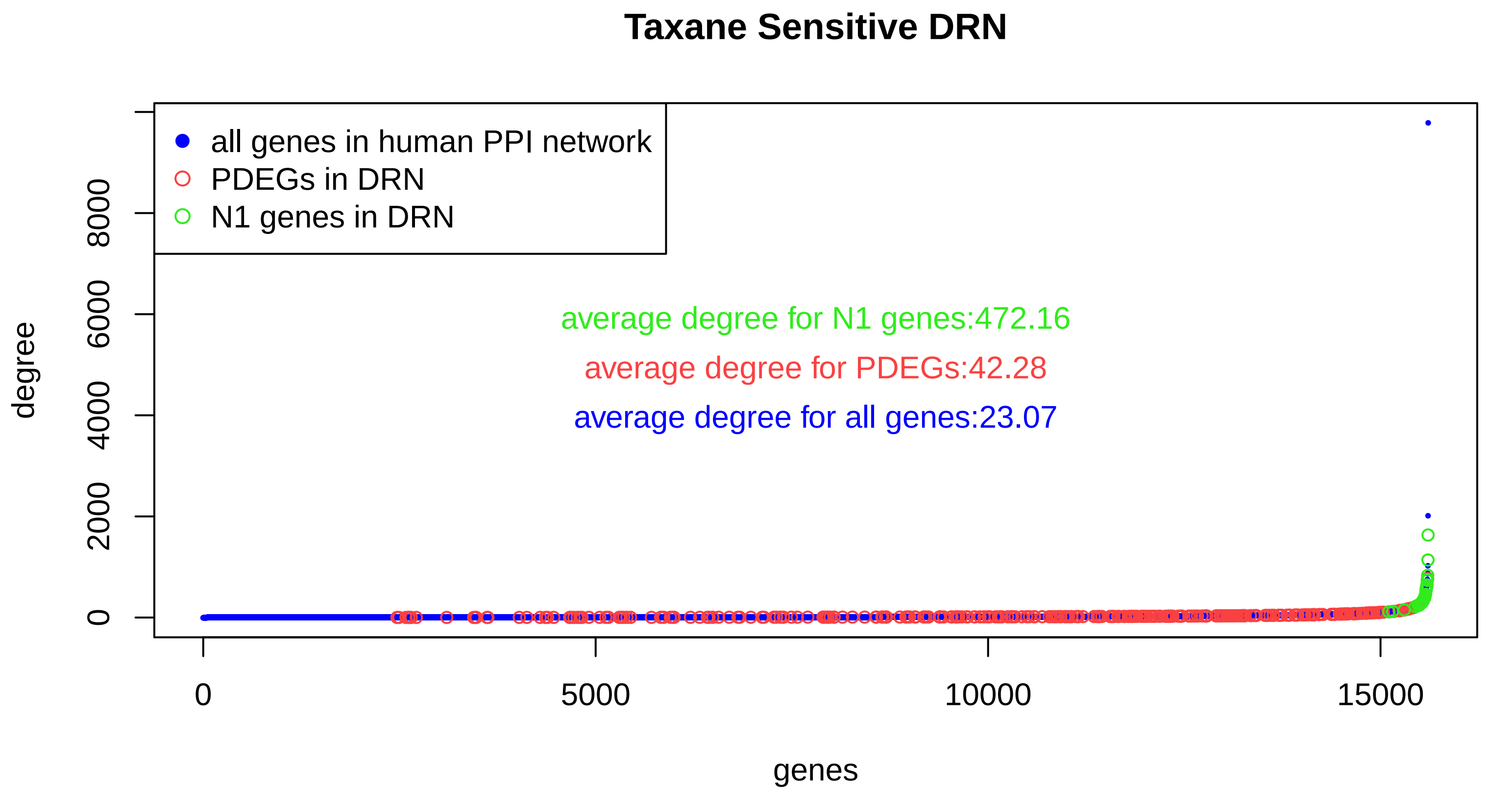

Taxane Sensitive DRN
all genes in human PPI network
PDEGs in DRN
N1 genes in DRN
average degree for N1 genes:472.16
average degree for PDEGs:42.28
average degree for all genes:23.07
degree
0
2000
4000
6000
8000
0
5000
10000
15000
genes
