# Supplementary figures and images for: A network-based phenotype mapping approach to identify genes that modulate drug response phenotypes
Source: Sci Rep. 2016 Nov 14;6:37003. doi: 10.1038/srep37003 (PMC5107984; doi:10.1038/srep37003)

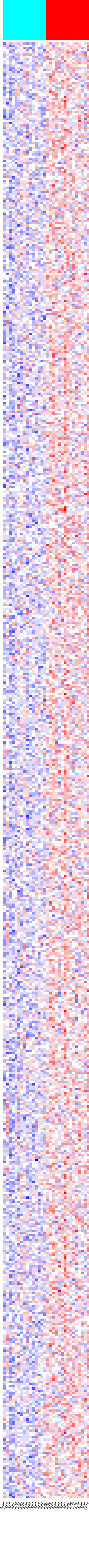

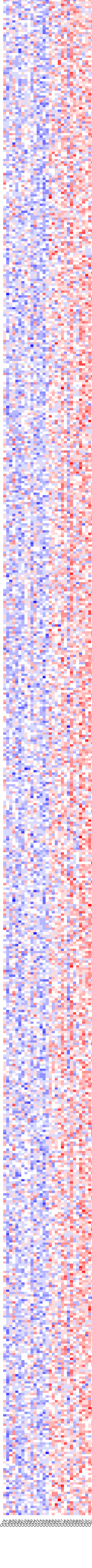



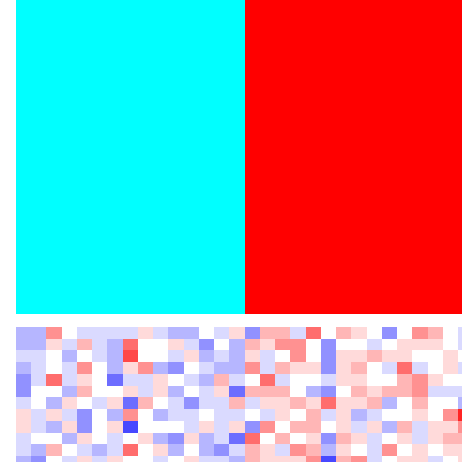

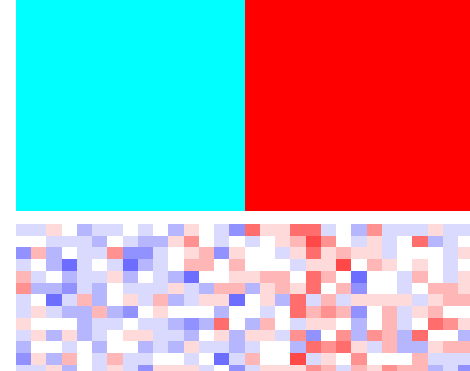

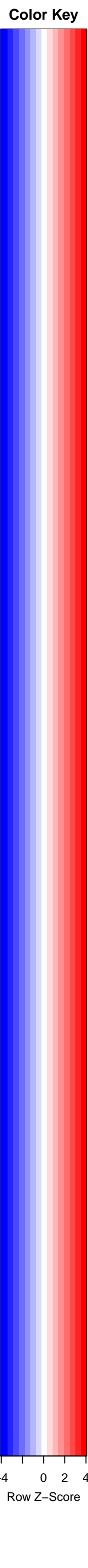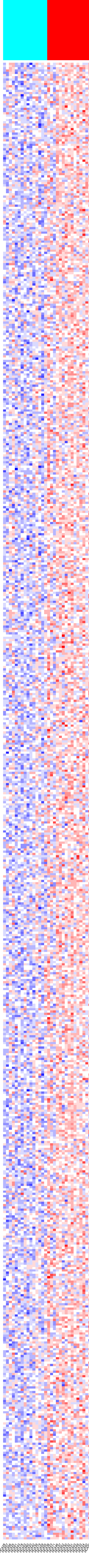

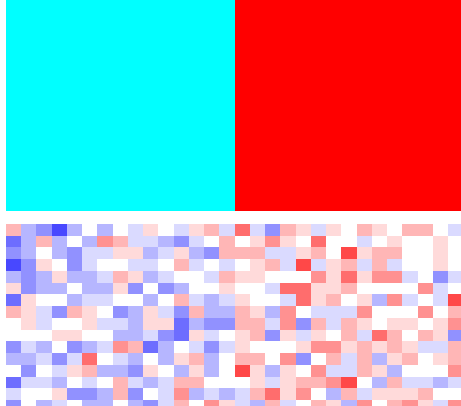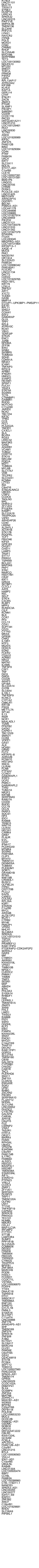







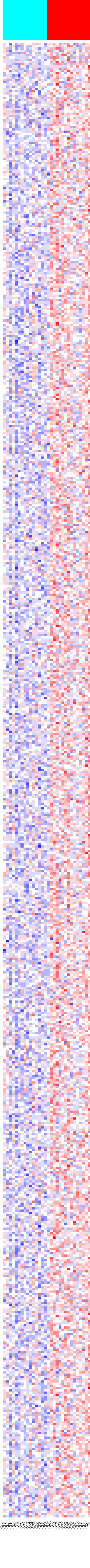







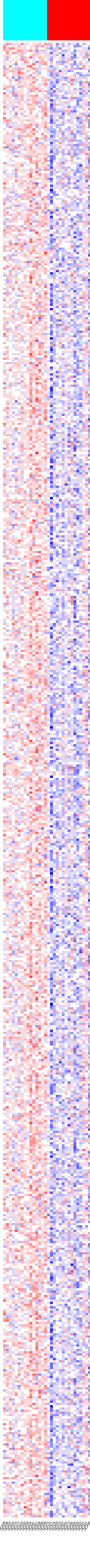







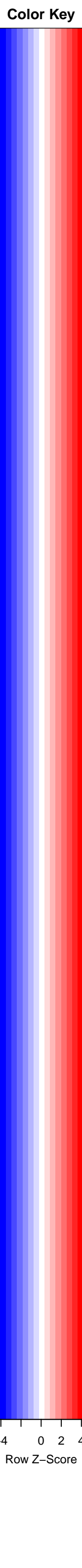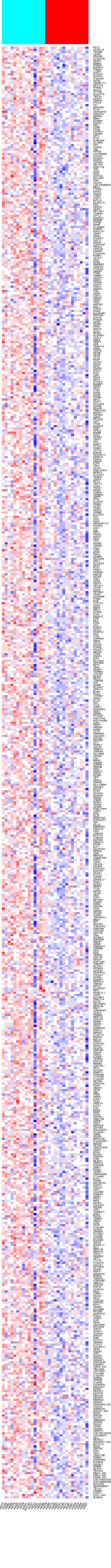





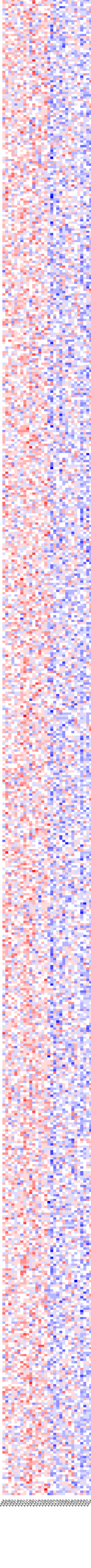

Supplement: Supplementary Information [file srep37003-s3.pdf]
